# Does Pay-For-Performance Program Increase Providers Adherence to Guidelines for Managing Hepatitis B and Hepatitis C Virus Infection in Taiwan?

**DOI:** 10.1371/journal.pone.0161002

**Published:** 2016-08-12

**Authors:** Huei-Ju Chen, Nicole Huang, Long-Sheng Chen, Yiing-Jenq Chou, Chung-Pin Li, Chen-Yi Wu, Yu-Chia Chang

**Affiliations:** 1 Institute of Hospital Health Care Administration, National Yang Ming University, Taipei, Taiwan; 2 National Health Insurance Administration, Ministry of Health and Welfare, Taipei, Taiwan; 3 Health Promotion Administration, Ministry of Health and Welfare, Taipei, Taiwan; 4 Institute of Public Health, National Yang Ming University, Taipei, Taiwan; 5 Department of Medicine, Taipei Veterans General Hospital, Taipei, Taiwan; 6 School of Medicine, National Yang Ming University, Taipei, Taiwan; 7 Department of Dermatology, Taipei Veterans General Hospital, Taipei, Taiwan; 8 Department of Healthcare Administration, Asia University, Taichung, Taiwan; 9 Department of Medical Research, China Medical University Hospital, China Medical University, Taichung, Taiwan; University of New South Wales, AUSTRALIA

## Abstract

**Background:**

Many people are concerned about that the quality of preventive care for patients with hepatitis B virus (HBV) and hepatitis C virus (HCV) infection is suboptimal. Taiwan, a hyperendemic area of chronic HBV and HCV infection, implemented a nationwide pay-for-performance (P4P) program in 2010, which aimed to improve the preventive care provided to HBV and HCV patients by increasing physicians’ adherence to guidelines through financial incentives. The objective of this study was to evaluate the early effects of the P4P program on utilization of preventive services by HBV and HCV patients.

**Methods:**

Using a quasi-experimental design with propensity score matching method, we matched the HBV and HCV patients enrolled in the P4P program with non-enrollees in 2010, resulting in 21,643 patients in each group. Generalized estimating equations was applied to examine the difference-in-difference effects of P4P program enrollment on the utilization of three guideline-recommended preventive services (regular outpatient follow-up visits, abdominal ultrasonography (US) examinations, and aspartate aminotransferase and alanine aminotransferase (AST/ALT) tests by HBV and HCV patients.

**Results:**

The P4P program enrollees were significantly more likely to attend twice-annual follow-up visits, to receive recommended US examinations and AST/ALT tests, than non-enrollees.

**Conclusions:**

The results of our preliminary assessment indicate that financial incentives offered by the P4P program was associated with a modest improvement in adherence to guidelines for better chronic HBV and HBC management.

## Introduction

Hepatocellular carcinoma (HCC) is the sixth most prevalent cancer and the third most frequent cause of cancer-related death worldwide [1–3]. Chronic hepatitis B virus (HBV) and hepatitis C virus (HCV) infections are well documented etiologic factors for liver cirrhosis, hepatic decompensation, and HCC [2–8]. Despite the availability of an effective HBV vaccination and strategies in avoiding viral transmission through contaminated blood against HCV infection [2, 3], an estimated 350 million and 180 million people are chronically infected with HBV and HCV, respectively, worldwide [9–12].

Preventing or deferring disease progression in chronically infected HBV and HCV patients has become a major clinical and policy priority. Although antiviral treatment is the most important factor to prevent HBV and HCV progression [13, 14], guidelines also highly recommended to strategies such as regular follow-up visits, abdominal ultrasonography (US) examinations, aspartate aminotransferase and alanine aminotransferase (AST/ALT) tests, and tests of viral load to improve the management of HBV and HCV patients [2, 10, 15, 16]. Studies have demonstrated that the appropriate management of HBV and HCV patients can result in shift to an earlier stage, asymptomatic cancer at diagnosis, and significantly increased survival from HCC [2, 3, 16–18].

Despite the establishment of guidelines, the quality of preventive care received by chronic HBV and HCV patients remains suboptimal. Studies have suggested that patients with chronic HBV do not receive adequate routine care, particularly regular follow-up visits and surveillance or testing [19–23]. Although many clinical decisions or guideline compliance are joint decisions between patients and providers, previous research has suggested that financial incentives to providers may play a major role in encouraging providers to adhere to such guidelines [24–27]. Numerous countries have adopted pay-for-performance (P4P) programs, which are modifications of traditional payment schemes to reward healthcare providers for desirable performance. P4P programs are heterogeneous in the targeting of healthcare providers and diseases or conditions, the type of incentive offered, and the type of quality indicator [24–27]. According to our medical literature search, no previous study has evaluated the effects of a P4P program on the provision of the recommended preventive services for chronic HBV and HCV routine care, particularly in settings where financial barriers to preventive services among patients are minimal.

HCC is a leading cause of cancer incidence and mortality in Taiwan [28], and the primary causes of HCC are HBV and HCV infection [29, 30]. In 2010, in order to improve appropriate management of patients chronically infected with HBV and HCV in ambulatory care settings, the National Health Insurance Administration (NHIA) in Taiwan implemented a P4P initiative specifically targeting providers’ performances for chronic HBV and HCV care (The HBV/HCV-P4P program). Based on clinical findings, practice guidelines, and expert consensus, the NHIA recommends a guideline of preventive care for chronic HBV and HCV patients. This P4P program aims to increase physicians’ adherence to the guideline for prescribing recommended preventive services to HBV and HCV patients through extra financial rewards. The P4P program requires physicians to provide routine preventive care to their patients, including twice-annual visits, abdominal US examinations, and AST and ALT laboratory tests [31]. Physicians receive extra financial rewards for patients who receive all of the recommended services. Thus, the aim of this study was to assess the association between financial incentives and providers’ performances for the quality of preventive care provided to HBV and HCV patients in Taiwan.

## Materials and Methods

### Background information

The National Health Insurance (NHI) is a mandatory single-payer social health insurance program implemented in Taiwan on March 1, 1995. More than 90% of all hospitals and clinics in Taiwan are contracted with the NHI to provide comprehensive medical care coverage, including ambulatory and inpatient care, dental services, rehabilitation, prescription drugs and others, to > 99% of the country’s 22.5 million residents [32–34]. A series of disease-specific P4P programs has been implemented in Taiwan since 2001, which predominantly target prevalent chronic diseases such as asthma, diabetes, and hypertension [32–34]. The HBV/HCV-P4P program is a voluntary program initiated in January 1, 2010, and is one of the most recent P4P initiatives [31]. Hospital or clinic physicians of internal medicine, family medicine, gastroenterology, and pediatrics are eligible to participate in this P4P program, and can recruit individual patients to the program. Patients with chronic HBV or HCV infection are eligible to participate.

In addition to regular reimbursement for healthcare services, the P4P program provides participating physicians additional case management fees if their patients complete an initial enrollment visit (New Taiwan (NT)$100/visit; U.S. Dollar (US$) 3/visit) and a maximum of two routine follow-up visits per year (NT$100/visit; US$ 3/visit). US examinations and AST/ALT tests are required in the initial and follow-up visits. Additional rewards are also provided for further screening, referral, and early detection of abnormalities (NT$500; US$ 15) and HCC (NT$1,000; US$ 30) [31].

### Data source and sample

This study had a quasi-experimental design using population-based data extracted from the NHI claims files from 2009 to 2011. The NHI claims files include the NHI enrollment files, claims data, medical personnel registry, and the hospital or clinic registry. Data related to the identification of individuals were encrypted before being released to the researchers, and personal privacy was therefore protected. This study was approved by the Institutional Review Board (IRB) of National Yang-Ming University (IRB No. YM103085E).

In this study, according to the P4P program’s eligibility criteria, we defined our sample of chronic HBV or HCV patients as individuals with ≥ two HBV- or HCV-related visits to the same providers during the 6 months prior to the index date. HBV and HCV infection were defined as the International Classification of Diseases, Ninth Revision, Clinical Modification (ICD-9-CM) codes 070.30–070.33, 070.51, 070.54, V02.61, and V02.62. The index date for each patient was defined as the date of the first recorded visit for chronic HBV or HCV in 2010. We excluded patients with HCC (ICD-9-CM code 155) or hepatic coma (ICD-9-CM codes 572.2, 070.20–070.22, 070.41, 070.44, 070.60, 070.71), patients aged < 20 years, patients with incomplete or missing data, and patients who died during the 1-year follow-up.

To minimize the potential influence of selection bias, we used two strategies for sample selection. First, among all eligible patients (n = 138,422), 39,976 HBV or HCV patients of the physicians who did not participate in the P4P program were excluded. By the end of the first year, 963 physicians had participated in the P4P program. Of all 98,446 chronic HBV/HCV patients managed by these physicians, 21,646 patients (22%) had enrolled in the P4P program. Because the enrollees might have differed from the non-enrollees, we used a propensity score matching (PSM) approach to match the enrollees and non-enrollees with similar baseline patient, physician and hospital characteristics [35]. The final sample included 43,286 patients with an equal number of patients in the P4P and non-P4P groups (n = 21,643).

### Independent/dependent variables

For this HBV/HCV P4P program, the NHIA developed a set of process-related quality measures to encourage the ongoing monitoring of HBV and HCV patients through regular follow-up visits, routine US screening, and AST/ALT tests. Three dependent variables were constructed in this study accordingly: (1) ≥ two HBV- or HCV-related outpatient follow-up visits per year, (2) ≥ two abdominal US examinations per year, and (3) ≥ two AST and ALT tests per year. The P4P program offers rewards for services provided by the same designated providers. Therefore, only the services provided by the same providers were included in subsequent analyses [31].

The primary independent variable was program enrollment. Using the NHI specific payment codes for this P4P program, the P4P program enrollees were defined as patients with payment coding from P4201C to P4205C. The remaining eligible patients were referred to as the non-enrollees.

Other covariates included for analysis were the characteristics of the patients, physician and hospitals. Patient characteristics included gender, age, ethnic status, socioeconomic status (SES), liver cirrhosis, number of chronic diseases, and any other mental and physical catastrophic illness. The age categories were 20–39 years, 40–59 years, and ≥ 60 years. The ethnic group categories were aborigine or nonaborigine. SES was defined as a patient’s own insurable wage if he or she was the insured or the insurable income of the insured if he or she was a dependent or did not have a clearly defined monthly wage. SES was classified into three categories. The NHI program is financed by wage-based premiums for people with a clearly-defined monthly wage, and by fixed premiums for people without a clearly-defined monthly wage. Therefore, patients with a clearly defined monthly wage were assigned to one of the three categories, in which high SES patients were defined as patients with an insurable wage ≥ NT$40,000 (US$ 1,250), middle SES patients were defined as patients with an insurable wage between NT$20,000 and NT$39,999 (US$ 625 and US$ 1,249), and low SES patients were defined as patients with an insurable wage < NT$20,000 (US$ 625). Patients without a clearly defined monthly wage were typically farmers, fishermen, or people with low income, and were included in the same low SES group as women with an insurable wage < NT$20,000 (US$ 625) [36]. The number of chronic diseases was calculated as the total count of the following 12 baseline comorbid conditions diagnosed by physicians: hypertension, diabetes, stroke, heart disease, dementia, carcinoma, arthropathy, chronic obstructive pulmonary disease, obesity, end-stage renal disease, human immunodeficiency virus infection, and hypercholesterolemia. The baseline comorbid conditions and liver cirrhosis were identified by having ≥ one hospital admission or ≥ three outpatient diagnoses of the disease or condition during the 12 months prior to the index date. Physician characteristics included gender, age (≥ 50 years or not), and specialty (internal medicine or not). Hospital characteristics was hospital or not (clinic).

### Statistical analysis

Patient, physician and hospital characteristics were compared between the program enrollees and non-enrollees using chi-square tests. For the PSM method, a multivariate logistic regression model that included patient, physician and hospital characteristics, and adherence pattern prior to the P4P program, was applied to obtain propensity scores for the probability of being in one of the two groups. We employed the caliper matching method (also known as the greedy algorithm), with one-to-one matching between the enrolled group and the comparison group based on the closest propensity score, repeating the process until the smaller group (the enrollees) had been exhausted [35, 37]. This matched sample was used in all subsequent analyses.

McNemar’s test was used to assess the significance of the differences between the repeated proportional outcome variables before and after the implementation of the P4P program. Then we adopted difference-in-difference (DD) methodology (a pre-post design with a control group) to compare the changes (or differences) in the outcome variables before and after the implementation of the P4P program between the enrolled group and the comparison group to derive the policy impact [38, 39]. The following equation was employed:

$$y=\beta_{0}+\beta_{1}Enrollee+\beta_{2}Time+\beta_{3}(Enrollee\times Time)+\mu$$

where y is the dependent variable. Enrollee is a dummy variable representing participation in the P4P program (Enrollee = 1). Time = 1 denoted the time period after policy implementation. The coefficient of Enrollee (*β*_1_) represents the difference in the outcome of interest between P4P group and non-P4P group before the program was implemented. The coefficient of Time (*β*_2_) represents change of non-P4P group in the different period. The coefficient of the interaction terms (*β*_3_) reflect the impact before and after the implementation of the P4P program between the P4P and non-P4P group.

To address the correlation between repeated observations in outcomes across time for the same patient, the multivariate logistic regression model with generalized estimating equations (GEE) method was applied to examine the DD effects of P4P enrollment on three process quality measures among the patients with chronic HBV or HCV. We also analyzed the effect of P4P enrollment on the overall provision of all three requirements. For these dichotomous outcome variables, we specified a binomial distribution with logit link and the correlation matrix was assumed to be unstructured. For each model, odds ratios (OR) and 95% confidence intervals (CI) were calculated. All analyses were performed using the statistical software package SAS Version 9.4 (SAS Institute, Inc, Cary, North Carolina, USA), and a P value < 0.05 was considered significant.

## Results

Table 1 lists the baseline characteristics of the patients with chronic HBV or HCV infection in the P4P and non-P4P groups of the unmatched and matched samples. Prior to the PSM process, the sample contained 98,446 eligible patients, including 21,646 (22%) in the P4P group and 76,800 (78%) in the non-P4P group. We detected significant differences in the majority of the patient, physician and hospital characteristics between the two groups in the unmatched sample (*P* < 0.001). After the PSM process, the P4P and non-P4P groups contained 43,286 matched patients, with 21,643 patients in each group. The two groups were similar in all observable characteristics.

**Table 1:** Characteristics of HBV/HCV patients for the unmatched and matched samples.

		Unmatched (N = 98,446)					1:1 Matched (N = 43,286)				
		P4P		Non-P4P			P4P		Non-P4P		
		N	%	N	%	*P*-value	N	%	N	%	*P*-value
**Total**		21646	100	76800	100		21643	100	21643	100	
**Gender**						< .0001					0.885
	Female	9562	44.2	32697	42.6		9560	44.2	9545	44.1	
	Male	12084	55.8	44103	57.4		12083	55.8	12098	55.9	
**Age**						< .0001					0.754
	20~39	5835	27.0	20113	26.2		5833	27.0	5860	27.1	
	40~59	11329	52.3	39641	51.6		11328	52.3	11364	52.5	
	≥ 60	4482	20.7	17046	22.2		4482	20.7	4419	20.4	
**Aborigine**						< .0001					0.750
	No	21231	98.1	75960	98.9		21231	98.1	21240	98.1	
	Yes	415	1.9	840	1.1		412	1.9	403	1.9	
**Socioeconomic status**						< .0001					0.934
	Low	7196	33.2	27302	35.6		7193	33.2	7208	33.3	
	Middle	8407	38.8	27399	35.7		8407	38.8	8370	38.7	
	High	6043	27.9	22099	28.8		6043	27.9	6065	28.0	
**Liver cirrhosis**						0.015					0.052
	No	21312	98.5	75783	98.7		21310	98.5	21358	98.7	
	Yes	334	1.5	1017	1.3		333	1.5	285	1.3	
**Catastrophic illness**						0.484					0.260
	No	20586	95.1	72949	95.0		20584	95.1	20634	95.3	
	Yes	1060	4.9	3851	5.0		1059	4.9	1009	4.7	
**Number of chronic diseases**						0.094					0.380
	0	13676	63.2	48942	63.7		13674	63.2	13791	63.7	
	1	4231	19.6	15070	19.6		4231	19.6	4216	19.5	
	≥ 2	3739	17.3	12788	16.7		3738	17.3	3636	16.8	
**≥ 2 follow-up visit during the preceding year**						< .0001					0.928
	No	7508	34.7	30772	40.1		7508	34.7	7499	34.7	
	Yes	14138	65.3	46028	59.9		14135	65.3	14144	65.4	
**Physician age**						< .0001					0.833
	<50	16196	74.8	51771	67.4		16194	74.8	16213	74.9	
	≥ 50	5450	25.2	25029	32.6		5449	25.2	5430	25.1	
**Specialty**						< .0001					0.891
	Internal medicine	18522	85.6	69530	90.5		18521	85.6	18531	85.6	
	Others	3124	14.4	7270	9.5		3122	14.4	3112	14.4	
**Hospital**						< .0001					0.802
	No (Clinical)	10000	46.2	18310	23.8		9997	46.2	10023	46.3	
	Yes	11646	53.8	58490	76.2		11646	53.8	11620	53.7	

Abbreviations: P4P, pay for performance

Table 2 presents the rates of attendance of follow-up visits, US examinations, and AST/ALT tests during the 1-year follow-up before and after the implementation of the P4P program. The proportion of patients receiving all three recommended services in the P4P group increased marginally from 25.0% to 25.2%. In the non-P4P group, the proportion of patients receiving all three services significant decreased from 22.7% to 21.0% (*P* < .0001). Specifically, the non-P4P group was associated with significant decrease in usage of regular follow-up visits (65.4% to 60.9%, *P* < .0001), US examination (26.1% to 24.0%, *P* < .0001), and AST/ALT tests (45.3% to 38.8%, *P* < .0001). On the other hand, the usage rates among patients in the P4P group either increased significantly in US examination *(P* = 0.017) or remained unchanged in regular follow up visits. The only exception was the decrease in usage of AST/ALT tests in the P4P group.

**Table 2:** The comparison of the proportions of patients who completed two recommended follow-up visits, US, and AST/ALT test in one year between the P4P.

		follow-up visits	US examinations	AST/ALT tests	All three services
**P4P group**					
	Before P4P	65.3%	27.7%	46.4%	25.0%
	After P4P	65.3%	28.5%	41.0%	25.2%
	*P*-value	1.000	0.017	< .0001	0.540
**Non-P4P group**					
	Before P4P	65.4%	26.1%	45.3%	22.7%
	After P4P	60.9%	24.0%	38.8%	21.0%
	*P*-value	< .0001	< .0001	< .0001	< .0001

Abbreviations: P4P, pay for performance; US, ultrasonography; AST, aspartate aminotransferase; ALT, alanine aminotransferase

Table 3 shows the results of DD estimates using a multivariate logistic regression model with GEE methods to compare process-related quality of care between the two study groups before and after the implementation of the P4P program. After controlling for individual, physician and hospital covariates, our results indicated statistically significant positive DD effects (Enrollee*Time). Following the introduction of the P4P program, the enrollees were significantly more likely to attend twice-annual visits (OR = 1.23; 95% CI: 1.17–1.29), and receive recommended US examinations (OR = 1.18; 95% CI: 1.12–1.24) and AST/ALT tests (OR = 1.05; 95% CI: 1.00–1.10), than were the non-enrollees. The enrollees (OR = 1.13; 95% CI: 1.07–1.19) were significantly more likely to receive all three recommended services than were the non-enrollees.

**Table 3:** Differences-in-differences (DD) estimates of multivariate logistic regression with GEE method of the impact of the P4P program on three recommended preventive processes for HBV/HCV patients (n = 43,286).

	follow-up visits		US examinations		AST/ALT tests		All three services	
	OR	(95% CI)	OR	(95% CI)	OR	(95% CI)	OR	(95% CI)
**Enrollee** (Ref: Non-P4P)								
P4P	1.00	(0.96–1.04)	1.10	(1.05–1.15)	1.06	(1.02–1.10)	1.16	(1.10–1.21)
**Time** (Ref: Before)								
After	0.82	(0.79–0.84)	0.89	(0.85–0.92)	0.75	(0.72–0.77)	0.90	(0.86–0.93)
Enrollee*Time								
DD	1.23	(1.17–1.29)	1.18	(1.12–1.24)	1.05	(1.00–1.10)	1.13	(1.07–1.19)

Extraneous factors adjusted in the model include gender, age, aborigine, socioeconomic status, liver cirrhosis, catastrophic illness, number of chronic diseases, physician gender, physician age, specialty, and hospital. Abbreviations: DD, differences-in-differences; GEE, generalized estimating equations; P4P, pay for performance; HBV, hepatitis B virus; HCV, hepatitis C virus; US, ultrasonography; AST, aspartate aminotransferase; ALT, alanine aminotransferase; OR, odds ratio; CI, confidence interval

## Discussion

The HBV/HCV P4P program predominantly aims to encourage the ongoing monitoring of chronic patients through regular follow-up visits, routine US screening, and AST/ALT tests. The results of this preliminary assessment indicate that whereas the non-P4P enrollees were associated with significant lower usage of all three recommended services than the P4P enrollees after the implementation of the P4P program, the magnitude of change difference between the two groups before and after the P4P program was modest (5%-23%).

P4P is an increasingly popular payment method for linking provider reimbursement to quality of care [24–27], a series of disease-specific P4P programs has been implemented in Taiwan since 2001 [32–34]. Although the effectiveness of P4P programs remains under debate [24–27], previous empirical findings in Taiwan suggest that the P4P programs in Taiwan are found to significantly increase physicians’ adherence to guidelines and positively contributes to the better management of patients with diabetes, breast cancer, or tuberculosis [40–44]. The positive effects of P4P strategies indicate that healthcare professionals can play a significant role in patient’s preventive service utilization, even for the measure of regular follow-up which is predominantly believed to be out-of physician’s reach. Some examples of physician’s actions may help to increase regular follow up, including telephone reminders from physician’s office, or involving case manager in coordinating the services. Additional financial incentives related to providers’ performances can increase provider adherence to the recommended guidelines for regular care for patients, and are later expected to improve the health outcomes of these patients. In the recently amended NHI Act, the expansion of P4P payment schemes is listed as a major policy goal. Accumulating evidence indicating the positive effects of the disease-oriented P4P programs supports the implementation of additional P4P programs in Taiwan.

In contrast to the previous findings in other P4P programs in Taiwan, the magnitude of change differences in utilization of recommended preventive services following the HBV/HCV-P4P program was relatively small. The results highlight two key potential reasons for the minor improvement observed. First, similar to the majority of P4P programs in several other countries, provider participation in the P4P program is voluntary and the participation rate tends to be low [27]. In the first year of the HBV/HCV-P4P program, only 18.7% of all eligible physicians participated (data not shown). Low participation from the targeted providers and small size of the bonuses are two commonly cited plausible explanations for either the modest findings or the absence of findings for an effect [27, 45]. In addition, recent research also indicates that the details of program design and implementation will also be critical to provider participation and behavioral change induced by P4P [46]. These details may include better communication between payers and providers regarding the nature of P4P interventions, the structuring and distribution of provider’s rewards, and the design and monitoring process of provider’s performance measurement. Performance metrics used need to be widely recognized in the targeted professional communities, and be regularly updated [25–27, 47]. More in-depth examinations of how the details of program designs, implementation, and management of P4P schemes should not be overlooked for further expansion.

Next, although a significantly greater number of P4P program enrollees received the guideline-recommended preventive services than did the non-enrollees, the rates of receiving all three recommended services in the P4P (25.2%) and non-P4P (21.0%) groups remained unsatisfactory, particularly for services such as US examination and AST/ALT tests. Due to poor compliance, the results probably represent the minimum improvement that can be expected from the P4P program. Our results are similar to those of previous studies, which indicated that adherence to HCC surveillance is suboptimal [19–21, 23, 48, 49]. In Taiwan, HBV and HCV infection are the leading cause of HCC [29, 30], these results suggest that providing financial incentives to healthcare professionals might be effective to some extent, but relying on a single P4P strategy to manage HBV and HCV patients might be insufficient. The financial barriers to the preventive services are minimal under the NHI program in Taiwan [32, 33]; therefore, other barriers to regular ultrasound and blood tests essential to the management of HBV and HCV patients, such as patients who are unaware of disease status, limited knowledge and understanding on disease, fear of stigmatization in society, and reluctance to receive undesired test results, require further investigation [19, 22].

On the other hand, whereas the usage rate of all three recommended services in the P4P group remained relatively unchanged after the HBV/HCV-P4P program implementation, the usage rate in the non-P4P group was associated with significant reductions. One plausible explanation is that the financial incentives may have led providers to preferentially follow those people where they receive the greatest payment and divert human resources and attention to some people over others. This may not be necessarily ideal for a population health intervention. Further research and health policy should pay more attention on this issue.

This study has a few limitations that should be noted. First, due to the voluntary nature of program participation, selection bias may be likely. To minimize the potential influence of selection bias, we used a PSM approach to match the enrollees and non-enrollees with similar baseline patient and provider characteristics [35, 37]. However, the PSM method cannot control for hidden biases that might be caused by other unobserved (such as health literacy) or unmeasured (such as education or marital status) variables. These unobservable confounders might lead to the erroneous estimation of the policy effect. Future research with randomized controlled design or availability of appropriate instrumental variables may help in this regard. Second, this study might suffer from certain inherent limitations because of the use of claims data. The NHI claims data does not include detailed clinical information such as viral load or laboratory test results, and data on the severity of conditions. Reliance on diagnoses to define comorbidities can also lead to possible misclassification. Third, because of the short duration of follow-up, we only analyzed process-related quality indicators. Additional studies with availability of data could assist to determine whether the P4P program can effectively prevent critical adverse patient outcomes, such as the exacerbation of HBV or HCV infection, the incidence of HCC, mortality. The cost-effectiveness test would be a useful later analysis for long term policy evaluation. Fourth, our study only included two observation time point, before and after the implementation of the P4P program, thus it’s hard to test the “parallel trends assumption”, inherent in DD analyses. But according to the theory about DD estimation, it has been proposed that the smaller the time period tested, the more likely the “parallel trends assumption” is to hold [50]. In terms of the influence of other external factors, no other obvious external factors, policies or events that may change or affect two groups differently during the two-year study period. However, we still cannot fully exclude the possibility. Fifth, we only included the regular follow-up visits, US examinations, and AST/ALT tests provided by the same designated providers for analysis. This may lead to possible underestimation of service uses if patients received these services from other providers during the study period. We conducted sensitivity analyses by including the services provided to each patient by different providers during the follow-ups and the results remained robust. Underestimation is likely to occur non-differentially between the P4P and non-P4P groups, so the true effects of the P4P program could be larger than observed. Finally, to ensure the same duration follow-up, we excluded 2,548 patients (1.8%) with HCC or hepatic coma, and who died during the 1-year follow-up. These patients would have been likely to have attended additional follow-up visits and undergone further screening and testing. Therefore, excluding these patients might have led to the underestimation of adherence to the guidelines.

According to our literature search, our study is the first nationwide population-based study to evaluate the early effects of a HBV/HCV-P4P program on the provision of recommended preventive services to patients under a universal insurance coverage system. Our large sample and comprehensive data are associated with sufficient statistical power to demonstrate a significant association between the P4P program and the quality of management of HBV and HCV patients during the first year of the program although the magnitude of differences was modest. We used PSM to match the P4P and non-P4P groups for individual and provider characteristics, which strengthens the validity of our findings [42]. We also used a DD analysis, which facilitates the comparison of the P4P and non-P4P groups before and after intervention, and might reduce the effects of preexisting differences to some extent. In the absence of a large-scale randomized controlled trail, the results from the quasi-experimental design may provide some valuable lessons about the effectiveness of the P4P intervention on utilization of preventive services among chronic HBV and HCV patients.

## Conclusions

Taiwan is a hyperendemic area of HBV/HCV infection [29], where low provision of the recommended preventive services poses a major clinical and public health concern. The modest positive effects of the first-year HBV/HCV-P4P program indicate that financial incentives may play a role to improve physicians’ adherence to guidelines and provide the prevention-related quality of care to chronic HBV or HCV patients. However, for further continuation and expansion of the P4P program for HBV/HCV patients or other medical conditions, more diligent efforts are required in improving physician’s participation, and randomized controlled trials and more dimensions of indicators including treatments and patient outcomes shall be carried out.
